# Primary Testicular Diffuse Large B-Cell Lymphoma: A Case Report

**DOI:** 10.4021/wjon629w

**Published:** 2013-03-06

**Authors:** Sebnem Izmr Guner, Didem Karacetin, Mahmut Yuksel

**Affiliations:** a Private Medicalpark Bahcelievler Hospital, Department of Hematology, Istanbul, Turkey; b Istanbul Research and Training Hospital, Radiation Oncology Department, Istanbul, Turkey; c Private Medicalpark Bahcelievler Hospital, Department of Nuclear Medicine, Istanbul, Turkey

**Keywords:** Testicular lymphoma, Germinal center, B-cell

## Abstract

Testicular lymphoma was first reported by Malassez and Curling in 1866. Primary testicular lymphoma constitutes only 1-7% of all testicular neoplasms and less than 1% of all non Hodgkin lymphoma. We report the case of a 47-year-old man without a particular past medical history, who presented with a painful left testicular swelling that he has noticed for several weeks. Radiological findings consisted in multiple hypoechoic masses that corresponded in histological examination to a diffuse intratubular lymphomatous infiltration situated away from the spermatic cord, the epididymis, ductuli efferentes and rete testis. Immunohistochemical study showed positivity of MUM-1, Bcl-2 and B-cell marker (CD20) and TdT, CD 3, CD5, Bcl-1, CD10, Bcl-6 and Myeloperoksidaz were negative. Ki-67 proliferation index was 90% of neoplastic lenfoid infiltration. The patient underwent full staging for lymphoma by positron emission tomography, showing right superior paratrakeal, precarinal, subcarinal, left paraaortic and retrocrural and left iliac involvement lymph nodes also the right testis and of extra-testicular involvement by the skeleton sistem. The diagnosis of stage III primary testicular large B-cell lymphoma of germinal center B-cell-like group was made. The patient is now treated by chemotherapy. Primary testicular lymphoma is a rare tumor whose diagnosis is based on histological findings. There are non consensual etiological or predisposing factors. Treatment modalities consist in surgical excision, chemotherapy and radiation therapy but the accurate procedures are not standardized. Factors that have been linked to more favorable outcomes include younger patient age, localized disease, presence of sclerosis at pathologic analysis, smaller tumor size, lower histological tumor grade and lack of epididymal or spermatic cord involvement.

## Introduction

Primary testicular lymphoma is a rare tumor accounting for 1% of all testicular non Hodgkin lymphoma [1]. It is defined by the primary localization of the tumor in the testis at presentation.

It accounts for approximately 1% of non-Hodgkin’s lymphoma, 4% of all extranodal non-Hodgkin’s lymphoma and 5% of all testicular malignancies with an estimated incidence of 0.26/100,000 per year [1-4]. Primary testicular lymphoma (PTL) is essentially an intermediate or high-grade lymphoma, and the diffuse large-cell type is the most common [5]. In secondary involvement of the testis other aggressive histologies are prevalent: in particular, Burkitt’s and Burkitt’s-like types have been reported in 10-20% of cases, chiefly in HIV+ patients. T-cell or follicular lymphomas involving the testes have been described in rare cases [6-10].

In contrast to other testicular malignancies, PTL occurs mainly in patients aged over 50 [10]. After adequate locoregional and systemic treatment the central nervous system (CNS) remains the most frequent site of recurrence (up to 30%). Prophylactic intrathecal (IT) chemotherapy (CT) combined with systemic treatment has therefore been introduced to improve outcome [1, 10, 11].

## Case Report

We report the case of a 47-year-old man without a particular past medical history, who presented with a painful right testicular swelling that he has noticed for several weeks. There was no reported history of trauma, night sweets, fever or chills. Scrotal examination revealed a firm and enlarged testis with a homolateral hydrocele. The remainder of the clinical examination was noncontributory. The ultra-sound examination showed an enlarged, heterogeneous testis with multiple hypoechoic masses. Laboratory tests, especially, the serum alpha-fetoprotein (αFP) and serum beta human chorionic gonadotropin (βHCG) levels were normal. Serum lactate dehydrogenase (LDH) was alittle bit high 294 U/L (125 - 243 U/L), Beta 2 Mikroglobulin was 2.18 mg/L (0.7 - 1.8 mg/L), CRP (C reactive protein) was 80.2 mg/L (0.8 - 2 mg/L), Sedimentation was 82 mm/h (0 - 20 mm/h). He has also a low anemia HGB was 13.0 g/dL (14.1 - 18.1 g/dL).The other laboratory tests were normal.

**Figure 1:**
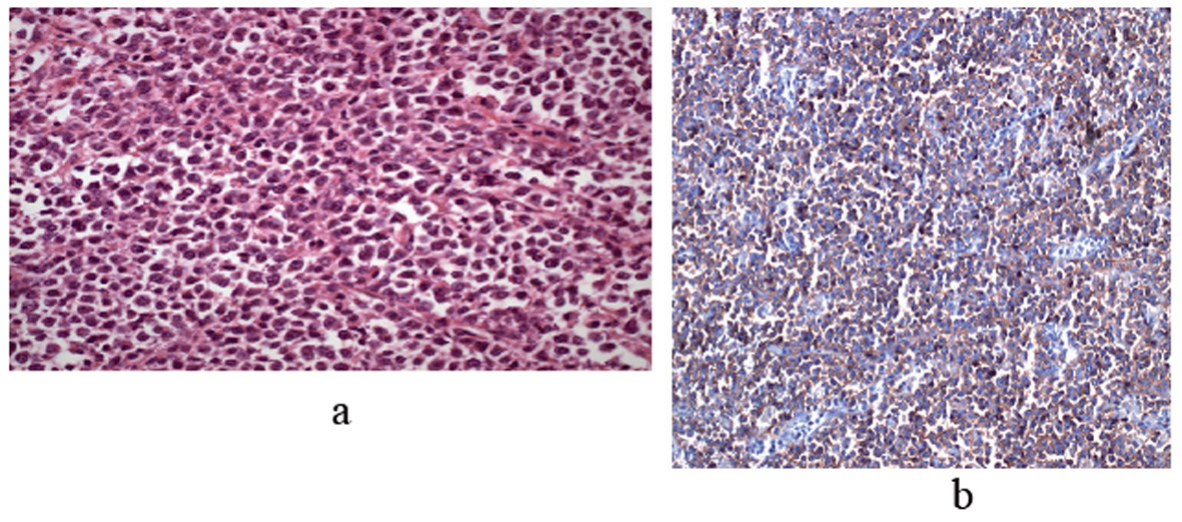
(a) Atypical lymphoid cells which are narrow cytoplasm, hyperchromatic enlarged nucleus which completely destroy normal testicular structure, (H&E × 400). (b) Diffuse membranose staining with CD20 in atypical lymphoid cells (CD20, × 200).

An excision of the right testis was performed. The light microscopy demonstrated a diffuse intratubular lymphomatous infiltration situated away from the spermatic cord, the epididymis, ductuli efferentes and rete testis. The malignant cells were large with scant cytoplasm and large vesicular nuclei (Fig. 1). The paraffin immunohistochemical staining showed positivity of B-cell marker (CD20), Bcl-2 and MUM-1. Tumor cells did not express CD10, Bcl-6 and Bcl -1 antigens.

**Figure 2:**
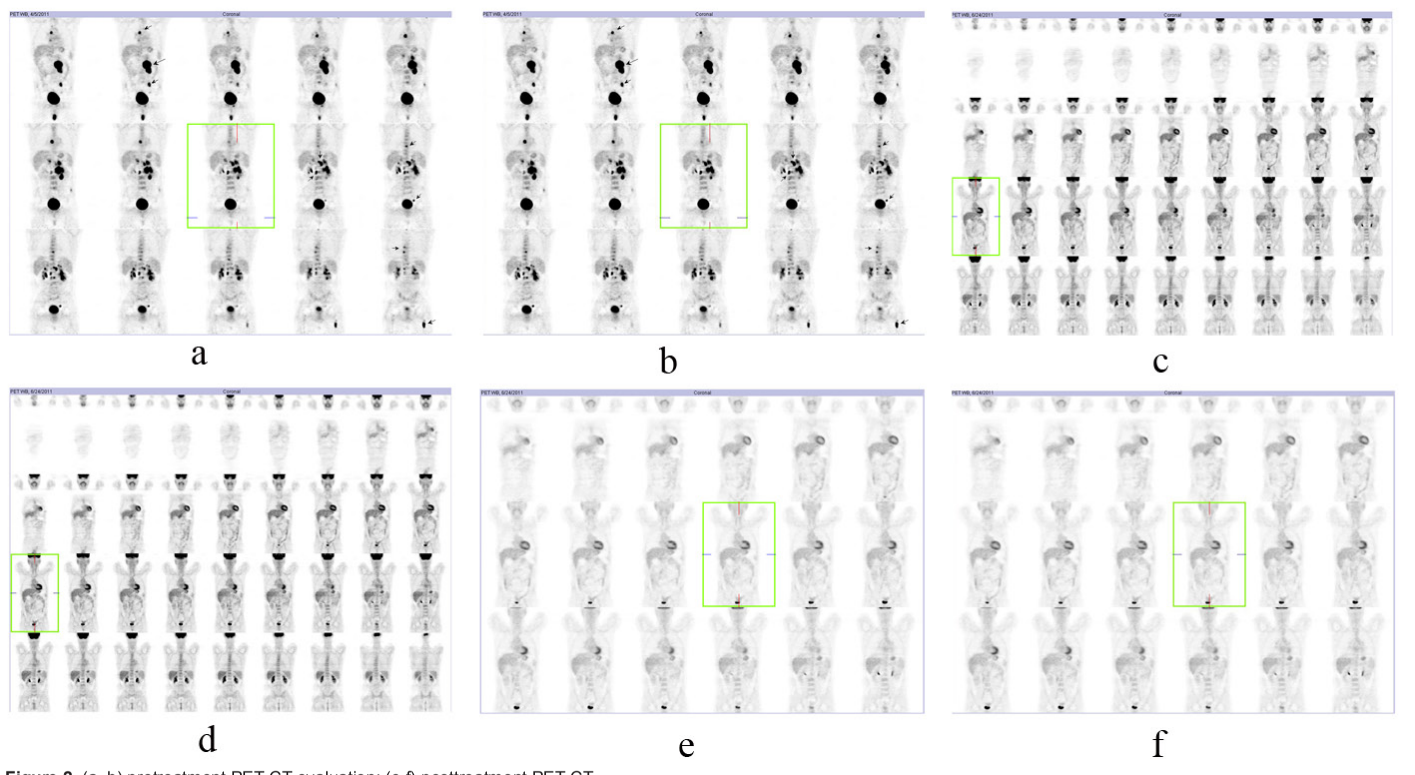
(a, b) pretreatment PET-CT evaluation; (c-f) posttreatment PET-CT.

The patient underwent full staging for lymphoma including of positron emission tomography, showing right superior paratrakeal, precarinal, subcarinal, left paraaortic and retrocrural and left iliac involvement lymph nodes also the right testis and of extra-testicular involvement by the bone system and bone marrow biopsy (Fig. 2a, b). The bone marrow biyopsi showed any involvement by lymphoma.

The diagnosis of stage III E primary testicular large B-cell lymphoma of germinal center-B-cell like group was made.

The patient was treated by chemotherapy of R-CHOP. After 8 cure chemotherapy the positron emission tomography was normal. Al laboratory tests were normal the patient was well. Complete remission was achieved in the patient (Fig. c-f).

Because of higher rates of relapse in the contra-lateral testis (up to 50% of patients) [10], prophylactic radiotherapy of 30 Gy was performed for the contralateral testis and central nervous system.

## Discussion

Testicular lymphoma was first reported by Malassez and Curling in 1866 [2, 3]. Primary testicular lymphoma constitutes only 1-7% of all testicular neoplasms and less than 1% of all non Hodgkin lymphoma [4]. The mean age at presentation is 60 years, but the recent published cases concerned patients younger than the past reported series and considered that this fact has a positive effect on the outcome of the patients [1, 5, 10]. According to the recent publications, our patient is young and is aged only 47 years. The typical presentation is a testicular painless mass of variable size that is usually unilateral [6, 10]. However, at presentation, a bilateral involvement is noticed in up to 10% of the cases [6]. Constitutional symptoms such as fever, weight loss, anorexia, night sweating and fatigue are seen in 25 to 40% of the patients [1-16].

Scrotal swelling, however, was not the presentation finding in our patient and he had no B symptoms such as fever and night sweating.

Primary testicular lymphoma has tendency to spread to several extra-nodal sites including the central nervous system (CNS), skin, lung, pleura, waldeyer’s ring, soft tissue and eyes [1, 7, 10].

The imaging features reflect its infiltrative but nondestructive characteristics. At ultra-sound examination, the normal homogeneous echogenic testis is replaced focally or diffusely with hypoechoic vascular lymphomatous tissue [8-10]. LDH levels have been correlated with tumor aggressiveness, whereas other tumor markers such as βHCG and αFP are rarely elevated in TNHL cases [4]. In our case, the βHCG and αFP levels were normal.

Histological examination is the only means of diagnosis. It can be made on biopsy or surgical specimen. Testicular lymphoma is locally aggressive and can typically infiltrate the epididymis, spermatic cord or scrotal skin [9]. The predominant histology is diffuse large B-cell lymphoma (DLBCL) [4]. It is reported in more than 70% of the cases [6]. The other sub-types include follicular lymphoma, plasmacytoma, lymphoblastic and Burkitt’s like lymphoma.

The DLBCL is classified as germinal center B-cell-like or non germinal center B-cell-like by means of immunohistochemical expression of CD10, bcl 6 and MUM1 [10]. The non-germinal center B-cell-like subgroup is the most frequent; it exhibits a high proliferative activity [4]. On the other hand, the germinal center B-cell type, like our reported case, is seen mostly in HIV-positive patients and has a better overall survival [6].

Histopathological differentiation of testicular lymphomas from germinal tumors is usually a challenge but these lymphomas generally appear more lobulated with well defined borders at ultra-sound examination [9]. Other conditions might mimic testicular lymphoma such as granulomatous orchitis, pseudolymphoma, palsmacytoma and rhabdomyosarcoma [4].

There are non consensual etiological or predisposing factors. Various reports have implicated prior trauma, chronic orchitis, cryptorchidism and filariasis of the spermatic cord as risk factors [9]. Treatment modalities consist in surgical excision, chemotherapy and radiation therapy, but the accurate procedures are not standardized.

Extranodal metastasis may be seen at the time of diagnosis or develop during clinical course of the disease. Most commonly involved sites are the central nervous system, Waldeyer’s ring, skin and the lungs, and prostate, however the kidneys, liver, bone marrow, pleura and bones are more rarely involved [11-16].

The most important factors determining the prognosis are stage and histological grade. Insufficient organ functions due to advanced age, presence of the constitutional symptoms, tumor burden higher than 9 cm, spermatic chord and bilateral testicular involvement, vascular invasion, degree of sclerosis and high level of LDH affects the prognosis negatively [11-16].

Of the known poor prognostic factors, our patient had involvement of the epididymis and spermatic chord.

Our patient was in stage III E according to Ann-Arborr staging system. According to the data of International Extranodal Lymphoma Working Group 57% of the cases with PTL is seen in stage I, 22% in stage II and 21% in stage III - IV [3].

Overall survival or disease-free survival is prolonged in this disease with combined chemotherapy with anthracycline (± radiation therapy) following orchiectomy in early stages.

Recently, combined modality treatment with systemic doxorubicine-based chemotherapy, prophylactic intrathecal chemotherapy and scrotal radiotherapy has been recommended because of the relapse risk at extra-nodal sites such as the CNS and contralateral testis.

Despite these more aggressive treatment modalities, prognosis is often poor, even in the localized disease with the two-year relapse rate exceeding 50%. Factors that have been linked to more favorable outcomes include younger patient age, localized disease, presence of sclerosis at pathologic analysis, smaller tumor size, lower histological tumor grade and lack of epididymal or spermatic cord involvement [9, 10]. According to these prognostic factors, our patient seems to have a good outcome because of his young age, the localized disease and the germinal center B-cell-like type tumor.

### Conclusion

In conclusion, primary testicular lymphoma is a rare disease and no consensus exists on its therapeutic modalities.
